# Perceptions of HIV patients on the use of cell phones as a tool to improve their antiretroviral adherence in Northwest, Ethiopia: a cross-sectional study

**DOI:** 10.1186/s12889-023-17452-3

**Published:** 2023-12-14

**Authors:** Sefefe Birhanu Tizie, Atsede Mazengia Shiferaw, Miftah Abdella Beshir, Muluken Belachew Mengistie, Sayih Mehari Degualem, Bayou Tilahun Assaye

**Affiliations:** 1 Department of Health Informatics, College of Health Sciences, Debre Markos University, Debre Markos, Ethiopia; 2 Department of Health Informatics College of Medicine and Health Science, University of Gondar, Gondar, Ethiopia; 3 School of Nursing, college of Medicine and Health science, Arbaminch University, Arbaminch, Ethiopia

**Keywords:** Adherence, HIV patient, Ethiopia, Perception, Use of mobile phone

## Abstract

**Background:**

Human immuno deficiency virus (HIV) is one of the most infectious diseases that cause death. A Medication non-adherence in HIV patient has been caused by factors such as not taking medications as prescribed by a physician, withdrawing from medication, missing appointments, and forgetfulness. To improve patients’ antiretroviral adherence, supporting them with mobile phone applications is advisable. This study aimed to assess HIV patients’ perceptions towards the use of cell phones to improve antiretroviral adherence.

**Methods and materials:**

An institutional-based cross-sectional study was conducted among 423 HIV patients at a comprehensive specialized hospital in northwest Ethiopia from June to July 2022. Study participants were selected using systematic random sampling techniques and the data collection tool was adopted and modified for different literatures. Data were collected through an online data collection tool, and STATA-14 software was used for analysis. Descriptive statistics and binary logistic regression were used. The variables with a P-value equal to or less than 0.2 in bivariable logistic regression were entered into a multivariable logistic regression, and model fitness was assessed.

**Results:**

A total of 410 study subjects have participated, making a response rate of 97%. In this study, 62% (95% CI: 57–67%) of HIV patients had a positive perception regarding the use of mobile phones to improve antiretroviral adherence. Perceived usefulness of mobile phones [AOR = 4.5, (95% CI: 2.2–9.1)], perceived ease of mobile phone use [AOR = 3.9, (95% CI: 2.0–7.5), age [AOR = 3.0, (95% CI: 1.5–6.2)], and educational status [AOR = 5.0, (95% CI: 2.3–10.0)] were significantly associated with HIV patients’ perception of mobile phones’ use to improve antiretroviral adherence.

**Conclusions:**

More than half of the respondents had positive perception regarding the use of mobile phones to enhance their adherence to treatment. Perceived usefulness, perceived ease of use, age, and educational status was significantly associated with perception of mobile phone use to enhance antiretroviral therapy adherence. Therefore, the government have to encourage and support patients in incorporating mobile phones into their antiretroviral therapy (ART) follow-up through training.

## Introduction

Drug and virologic rebound resistance are closely correlated with non-adherence to antiretroviral treatment [1]. More than 50% of all medications are prescribed, delivered, or sold improperly, and more than half of patients do not properly take their medications [2]. Although human immunodeficiency virus (HIV) infection has shifted from a terrible and deadly sickness to a more readily treatable disease, the combination of medications has radically changed the lives of people living with HIV/AIDS [3, 4].

Despite strategies being implemented to reduce new infections and deaths from HIV, Sub-Saharan Africa has an extremely high burden of HIV infection, accounting for more than 70% of the worldwide high load [5].

In Ethiopia, HIV/AIDS has been ongoing and epidemic as usual, with a prevalence rate of 0.9% [6, 7]. The strategies used for reducing the prevalence of HIV are the antiretroviral therapy treatments, if they are supported by technology, which will be significantly important for better treatment adherence. As scholars suggested, if patients started their treatments early, they would be regulated based on the permission of health professionals who would closely follow up, the quality of care would be high, and the life expectancy would be increased, like that of a person living free from HIV [8, 9].

Today, mobile phones are essential information tools for people in all aspects of life. Globally, mobile devices are transforming the delivery of health care [10]. People living with HIV/AIDS use technology to meet their health information needs. The use of mobile health is a viable tool in HIV treatment, and it is appropriate for People Living With HIV [11]. Mobile phone technologies have the potential to promote adherence, including wireless telecommunications networks; Sending and receiving messages on the wireless mobile telephone has become an extremely popular means of communication in the management of health conditions [12–14].

Mobile phone-based interventions are significantly important to achieving the 95-95-95 strategy and effectively implementing it, leading to increased HIV testing, improved ART adherence, and enhanced viral suppression rates. Mobile phones, like text message campaigns and mobile apps, can disseminate accurate and up-to-date information that can be used to raise awareness about HIV, promote HIV testing and counseling, and provide information on testing locations and services. It is also effective in improving adherence and retention rates. Mobile-based platforms can enable healthcare providers to remotely monitor viral load and treatment progress, patients’ adherence to ART, detect treatment failures, and provide timely interventions. This helps provide additional support to achieve viral suppression [15].

In the healthcare system, mobile health has a means of supporting treatment adherence since medication non-adherence has been caused by factors such as not taking medications as prescribed by a professional, withdrawing from medication, losing appointments, and forgetfulness [16].To support patients using technology like different mobile health programs, it is important to identify a user’s perception of the use of a mobile phone [2]. To the extent of the researcher’s search skills, no study has been conducted in Ethiopia, this study fills the gap regarding the perception of mobile phone use to support antiretroviral treatment adherence among HIV-patients attending ART clinics. Therefore, the study aimed to assess HIV patient’s perceptions towards the use of cell phones to improve antiretroviral adherence.

## Methods and materials

### Study design and setting

An institutional-based cross-sectional study was conducted at the University of Gondar comprehensive specialized teaching hospital from June to July 2022. The hospital is located in the northwest, 741 km from Addis Ababa, Ethiopia’s capital city. It is one of the biggest tertiary teaching and specialized hospitals. Currently, the hospital serves more than 15,939 HIV patients who have received ART services from the catchment areas.

### Source and study populations

#### Source population

All adult HIV patients who have ART follow-up at the University Of Gondar comprehensive specialized Hospital.

#### Study population

All HIV patients whose age is ≥ 18 years and who have ART follow-up from June to July 2022 at the University of Gondar comprehensive specialized hospital.

#### Sample population

Selected HIV patients whose age is ≥ 18 years and who have ART follow-up from June to July 2022 at the University of Gondar Hospital.

### Sample size determination and sampling procedure

The sample size was determined using the single population proportion formula by considering the 95% confidence interval, 5% of marginal error (d = 0.05), and 50% of the magnitude of perception since there has been no previous study done in the same population among HIV patients (*p* = 0.5). After accounting for the 10% non-response rate, the total final sample size was 423. A systematic random sampling technique was used to select study participants. The expected number of patients coming during the data collection period was 1256, which was obtained from Gondar Specialized Teaching Hospital. An interval was calculated, as k = N/n N = Expected number of HIV in ART follow up during data collection period n = sample of HIV patients (423), K = 1256/423 = 3 after that, within an interval of three HIV patients selected based on their order of registration and continuing until the sample was reached.

### Inclusion and exclusion

All HIV patients who had follow-up from June to July 2022 at the University of Gondar comprehensive specialized hospital were included in our study, and those HIV patients who were seriously ill and had a mental disorder during the data collection period were excluded from the study.

### Study variables

#### Dependent variable

perception to use of mobile phone.

#### Independent variable

socio dimorphic factors, perceived usefulness of mobile phone, perceived ease of use of mobile phone, mobile use pattern, environmental factors, clinical, and behavioral factors.

### Operational definitions

#### Perception to use a mobile phone

The study participants who scored above the mean on the 5-point Likert scale were categorized had a good perception, while those who scored below the mean had a poor perception [17–20].

#### Perceived usefulness

The study participants who scored above the mean on the 5-point Likert scale items were categorized as those who believed mobile phones were useful for their adherence, and those who scored below the mean were categorized as thinking mobile phone support was not useful for their adherence [21].

#### Perceived ease of use

The study participants who scored above the mean on the 5-point Likert scale items were categorized as thinking mobile phones were easy to use, and those who scored below the mean were categorized as thinking mobile phones were not easy to use [21].

### Data collection tools

A structured administrative questionnaire was adapted and modified from various Literatures. The content validity of the questionnaire was checked, and the reliability was calculated using Cronbach’s alpha coefficient (= 0.74), which was good. The data were collected based on the study participants’ (socio-demographic factors, environmental factors, behavioral factors, perceived usefulness, perceived ease of use, mobile phone use pattern) as independent variables, and perception of regarding mobile phone use as dependent variable [18–20, 22–28]. The questionnaire was first prepared in English, and then translated into the local Amharic language and back into English by experts to ensure consistency.

### Data quality assurance

A pre-test was conducted in the Tibebe Gihon comprehensive specialized teaching hospital among 10% of HIV patients. Before the actual data collection, modifications were made based on the pre-test. Two BSC nurses and one health informatics professional participated as data collectors and supervisors, respectively. The data collectors and the supervisor were trained before participating in the actual data collection process. To create awareness of the purpose of the study, their rights, and confidentiality issues, sufficient time was given to respondents to read and fill in materials carefully. There was continuous supervision up to the end of data collection. After collecting the data, the supervisor and the investigator checked its consistency and completeness.

### Data management and analysis

The data were downloaded since the data were collected by Kobo Collect Toolbox and exported to STATA 14 for statistical analysis and to generate descriptive statistics of the collected data to describe variables in the study using statistical measurements. The likert scale of the outcome variable were analysis by using 11 items (1;strongly disagree,2;disagree ,3;neutral, 4; agree, 5;strongly agree). A bivariable logistic regression was performed to identify factors associated with individual variables. Variables with a p-value less than or equal to 0.2 from the bivariable analysis were used for the multivariable analysis. A significant association was interpreted using an odds ratio, a 95% confidence interval, and a p-value less than 0.05. The Hosmer-Lemeshow test was used to test model fitness, and it was a good fit. Multicollinearity was checked using variance inflation factors (VIF) between independent variables, and there is no multicollinearity.

## Results

### Socio-demographic characteristics

A total of 97% of HIV patients correctly responded to this study. The mean age of the study participants was 37.4 ± 0.59 (SD) years. Approximately 258 (63%) of the study participants were female; regarding the educational status of study participants, 72% were educated; and in terms of residence, 70% of respondents were from urban areas. On the other hand, 82% of respondents were married (Table 1).

**Table 1:** Socio demographic characteristics of HIV patients towards perceptions of mobile phone use for ART adherence at University of Gondar specialized teaching hospital, Ethiopia, 2022(n = 410)

*Variable*	*Category*	*Frequency*	*Percent*
Sex	Male	152	37.02
	Female	258	62.93
Age	<=30 years	156	38.05
	> 30 years	254	61.95
Residence	Urban	289	70.49
	Rural	121	29.51
Religious status	Orthodox	312	76.10
	Muslim	78	19.02
	Other	20	4.88
Marital status	Unmarried	73	17.80
	Married	337	82.20
Educational status	Formal education	116	28.29
	No formal education	294	71.71
Employment status	Employed	248	60.49
	Unemployed	162	39.51

### Environmental factors

86.34% of the study participants traveled from home to the hospital with the use of public transport; 62.93% of the participants spent at least 1 US dollar monthly on transportation to visit the hospital; 71.46% traveled at least one hour from home to the hospital to access health services (Table 2).

**Table 2:** Environmental factors on perceptions of HIV patients to use mobile phone for ART adherence at university of Gondar specialized teaching hospital, Ethiopia (n = 410)

*Variable*	*Category*	*Frequency*	*Percent*
Distance	≤ 10 km	138	33.66
	> 10 km	272	66.34
Transportation	Public transport	354	86.34
	Car (own)	21	5.12
	Walk	35	8.54
Cost of transportation	≥ 1 US dollar	258	62.93
	< 1 US dollar	99	24.15
	No	53	12.92
Travel time	≥ 1 h	293	71.46
	< 1 h	117	28.54

### Mobile phone use pattern

In this study, a total of 83% of participants reported owning a mobile phone and being able to send, receive, and read messages. Additionally, 86% of participants can afford a cell phone, and 82% use an alarm. Among these users, 41.71% use alarms to wake up, and 35.8% use them to remind patients to take their medications. The study found that the majority of HIV patients anticipated problems related to mobile phone confidentiality (28.2%) and language barriers (41.22%). Interestingly, 17.6% of patients did not use an alarm, and most of the study participants preferred voice calls (49.02%). Furthermore, 48.78% of participants shared their mobile phones with others. On the other hand, out of the total number of study participants, 54% perceived mobile phones as useful, and about 45% thought that mobile phones were easy to use (Table 3).

**Table 3:** Mobile phone use pattern of HIV patients for ART adherence at University of Gondar comprehensive specialized teaching hospitals, Ethiopia, 2022 (n = 410)

*Variable*	*Category*	*Frequency*	*Percent*
Mobile phone ownership	Yes	341	83.17
	No	69	16.83
Can send or read message	Yes	284	83.2
	No	57	16.8
Affordability	Yes	283	69
	No	121	31
Alarm use	Yes	338	82.4
	No	72	17.6
Purpose of alarm	To wake up	171	41.7
	To remind appointments	20	4.8
	To remind my medications	147	35.9
	No use of Alarm	72	17.6
problem to use mobile Phone	Yes	410	100
Type of problem	Affect Confidentiality	116	28.29
	Language Problem	108	26.34
	Network	169	41.22
	Other	17	4.15
Langue barrier	English	359	87.56
	Amharic	51	12.44
Internet use	Yes	79	19.27
	No	331	80.73
Communication mood	Voice call	201	49.02
	Text	51	12.44
	both Text and voice	72	17.56
	No	86	20.98
Password use	Yes	161	39.27
	No	249	60.73
Mobile phone use with others	Yes	200	48.78
	No	210	51.22
Cellphone left, damaged before	Yes	176	42.93
	No	234	57.07
Usefulness of mobile phone	Useful	221	53.9
	Not useful	189	46.10
Ease of use of mobile phone	Easy	186	45.37
	Not easy	224	54.63

### Clinical and behavioral factors

The study revealed that the majority of participants (61.95%) utilize mobile phones as a means to decrease the number of cases within the community and to avoid experiencing stigma and discrimination. Also, 60.73% of patients disclosed their HIV status to their families. Besides this, a significant number of participants (59.76%) failed to take a dose in the preceding 6 months, with forgetfulness (36.59%) being the most prevalent reason. Furthermore, 31.22% of patients missed appointments in the last six months due to inadequate follow-up (Table 4).

**Table 4:** Clinical and behavioral factors of HIV patients to use mobile phone for ART adherence at University of Gondar specialized teaching hospitals, Ethiopia, 2022 (n = 410)

*Variable*	*Category*	*Frequency*	*Percent*
Stigma and discrimination	Yes	254	61.95
	No	156	38.05
HIV disclosure status	Yes	249	60.73
	No	161	39.27
Missing to take dose in last 6 month	Yes	245	59.76
	No	165	40.2
Reason for missing dose	Forget about it	150	36.59
	Busy with another thing	32	7.80
	Other	65	15.8
	No	163	40.16
Missed appointment in last 6 month	Yes	172	41.95
	No	238	58.05
Reason for missed appointment	Away from home	128	31.22
	reserved medication	44	10.73
	No	238	58.05

### **Perception of using a mobile phone**

In this study, a total of 62% of HIV patients had a positive perception regarding the use of mobile phones (95% CI, 57–67%). Specifically, educated patients and the younger age group demonstrated a favorable perception of using mobile phones for treatment adherence. Furthermore, it was observed that a majority of patients recognized the usefulness of mobile phones in ensuring proper adherence to their treatment regimen.

### Factors associated with the perception to use a mobile phone for improve HIV patient treatment adherence

The bivariate analysis shows that several variables, including sex, age, educational status, employment status, affordability, type of problem, communication mood, mobile use with family, perceived usefulness, and perceived ease of use, were found to have significant associations in the logistic regression analysis. However, in the multivariable analysis, it was determined that perceived usefulness, perceived ease of use, age, and educational status were the key factors significantly associated with the outcome. Based on this finding, patients who perceived mobile support as useful were 4.5 times more likely to have a positive perception of it compared to those who did not perceive using the mobile phone as useful [AOR = 4.5, (95% CI: 2.2–9.1)]. Patients who perceived mobile support as easy to use were four times more likely to experience the desired outcome compared to those who perceived mobile phone ease of use differently [AOR = 3.9, (95% CI: 2.0–7.5). A patient under 30 years old was perceived by respondents to be three times more likely to use a mobile phone than a patient above 30 years old age = < 30 years [AOR = 3.0, (95% CI: 1.5–6.2)]. From the respondent: Educated patients were perceived to be five times more likely to use a mobile phone than uneducated patients and educational status thought Educated [AOR = 5.0, (95% CI: 2.3–10.0)], *(*Table 5*)*.

**Table 5:** Binary and multivariable logistic regression analysis of perception of HIV patients to use mobile phone support for ART adherence at University of Gondar specialized teaching hospitals, Ethiopia, 2022 (n = 410)

*Variables*	*Variables Categories*	*Perception*		*COR (95%CI)*	*AOR (95%CI)*	*p-value*
		**Good (%)**	**Poor (%)**			
Sex	Female	180(43.0)	76(18.54)	2.3(1.5–3.5)	1.2(0.6–2.3)	0.57
	Male	76(18.54)	78(19.02)	1	1	
Age	=< 30 years	133(32.4)	123(30)	4.5(2.8–7.1)	3.0(1.5–6.2) **	0.019
	> 30 years	30(7.32)	124(30.24)	1	1	
Marital status	Married	199(48.4)	138(33.66)	0.41(0.2–0.7)	1.5(0.6–3.8)	0.92
	Unmarried	57(13.90)	16(3.90)	1	1	
Educational status	Formal education	227(55.7)	67(16.34)	10.2(6.2–16.8)	5.0(2.3–10.9) ***	0.00
	No formal education	29(7.07)	87(21.22)	1	1	
Employment status	employed	107(26.1)	141(34.9)	0.54(0.4–0.8)	0.6(0.2–1.3 )	1.55
	Unemployed	115(28.5)	47(11.46)	1	1	
Affordability	Yes	237(57.0)	116(28.29)	4.1(2.3–7.4)	1.5(0.4–5.1)	0.552
	No	19(4.63)	38(9.27)	1	1	
Type of problem	Confidentiality	89(21.71)	25(6.10)	5.1(2.6–9.8)	2.3(0.7–8.2)	
	Language Problem	66(16.10)	70(17.07)	5.5(2.7–11.0)	2.7(0.8-9.0)	0.12
	Network	73(17.80)	19(4.63)	1.4(0.8–2.4)	0.7(0.2–2.2)	0.5
	Other	28(6.83)	40(9.76)	1	1	
Communication mood	Voice call	133(32.44)	57(13.90)	3.6(2.2–5.8)	1.7(0.8–3.4)	
	Text	44(10.73)	12(2.93)	5.6(2.7–11.7)	1.6(0.6–4.6)	0.4
	Text and voice	33(8.05)	15(3.66)	3.3(1.6–6.8)	2.5(0.9–7.3)	0.8
	No	46(11.22)	70(17.07)	1	1	
Cell phone use with family	Yes	151(36.3)	49(12.0)	3.1(2.0-4.7)	1.7(0.9-2.0)	0.096
	No	105(25.6)	105(25.1)	1	1	
Usefulness of mobile phone	Useful	167(40.7)	22(5.37)	11.3(6.7–18.9)	4.5(2.2–9.1) ***	0.01
	not useful	89(21.7)	132(32.02)	1	1	
Ease of use mobile phone	Easy	189(46.1)	39(9.51)	8.3(5.3–13.1)	3.9(2.0-7.5) ***	0.004
	Not Easy	67(16.34)	115(28.05)	1	1	

## Discussion

To improve ART adherence for lifelong treatment of HIV patients, supporting patients with mobile technology is critical to ensuring the proper use and taking of drugs by respecting appointments based on the permission of health professionals.

Key findings from this study showed (62%) of participants had a good perception of use of mobile phones (95% CI, 57–67%). Studies conducted in different settings, South Africa [29], Peru [30] and the United States [31] were higher as compared with the current study. But, the result from this study is in line with the studies conducted in Uganda [32, 33], North West Ethiopia [2], Kenya, Germany, and India [20, 23, 27, 34]. The differences might be due to the advances in information and communication technology (ICT) infrastructure. The finding also shows most patients were able to read a short text message on their mobile phone. This finding is higher than the study conducted in North West Ethiopia at University of Gondar Hospital (72.2%) [2]. The disparity might be due to the literacy rate and economic status of the countries.

Regarding to the perceived the usefulness of mobile phone support almost half of the study participants respond not useful to perceive the use of the mobile phone for ART adherence. This finding is lower than the study conducted in Ghana even the study shows mobile users have a positive association with the use of mobile support for medication adherence compared to non-users in Nigeria [22, 35]. However the study’s results was supported by the study conducted in Pakistan, Uganda, South Africa, and Germany, which found that SMS reminders were useful for treatment adherence [23, 28, 36–39]. As research indicated, if patients had positive perceptions of the usefulness of mobile phones, their adherence to ART was more likely to be supported as compared to those who did not perceive the usefulness of mobile phones.

The other major factor that affects the perception of HIV patient adherence is the perceived ease of use of mobile phones. Based on the study results, more than half of the study participants responded that it was not easy to use the mobile phone. It was supported by the study conducted in Mozambique and Uganda, which showed that mobile devices are not easy to use [26, 37, 39]. We speculate that, although the participants do not understand the content of the message, they intend to use it as an alarm to take ART medication or obtain assistance from people around them. Studies conducted in China and North West Ethiopia published that a higher proportion of patients than ours were willing to accept support from the ART Clinic [2, 40]. This discrepancy might be due to the difference in fast-growing mobile phone technology, the need for multipurpose mobile phones, and a better understanding of the usefulness of mobile phone. Their practical level of use is easy due to the advancement of mobile devices, especially voice calls, which are the most commonly used and preferred way of communication among HIV patients that helps to maintain confidentiality.

The result of this study also indicated that patients whose age was less than thirty years (62%) had a better perception than their older counterparts. The study was supported by a study conducted in Kenya, Afghanistan, South Africa, and Germany [2, 17, 20, 23, 24]. According to different scholars, young people are more familiar with using mobile phone technology than other age groups since they are more vulnerable in their lives.

Different studies revealed that the age of the patient is correlated with the perception of mobile phones better among younger PLWH than their older counterparts [2, 40]. The findings of our study support this fact. This might be due to the early adopters’ better understanding of the technology among the younger generation.

According to this research result, 72% of educated patients had better perceptions of using mobile phones for ART adherence than uneducated patients. The study is supported by Afghanistan and South Africa [17, 24]. The literate were 3.6 times more likely to be open to receiving health-related information on mobile phones. It might be that developing countries have increased their mobile penetration from time to time, and mobile phones have integrated the daily lives of many people, including educated patients who can access medical information, make appointments, and set alarms since they are easier to use practically than for the uneducated.

Sharing mobile phones with family members and friends was found to be a challenge since text messages can disclose HIV status. Patients who disclosed their HIV status were more likely to have a positive perception of the ART clinic when compared to those who did not disclose their status. This might be because there is still a high stigma and discrimination in the community. This finding is similar to a study from Uganda and South Africa [29, 33]. This may be due to low HIV status disclosure and a fear of stigma and discrimination. Nowadays, everybody has a mobile phone. The likelihood of perceiving that you can improve ART adherence by supporting it with their mobile phone from the ART Clinic was higher among patients who perceived that their mobile phone could support their ART adherence than among those who did not perceive that SMS could support their adherence.

### Strengths and limitations

Since this study is the first in the study setting, it can be used as a baseline study, and it identifies factors affecting the perception of using mobile phones to improve treatment adherence among HIV patients. It was a cross-sectional study, so it may not articulate causal effects among different variables. Due to the limited research done before the study sitting, it was difficult to compare and contrast the findings as per our searching skills, and it would have been preferable if other researchers would have supported it qualitatively.

#### Conclusions

More than half of the respondents had a positive perception of using mobile phones to enhance their adherence to treatment. The perception was significantly associated with factors such as perceived usefulness, perceived ease of use, age, and educational status. The government further encourages and supports patients in incorporating mobile phones into their antiretroviral therapy (ART) follow-up. This can be accomplished by offering training and investing resources with the intention of helping HIV patients use mobile phones efficiently for their treatment.

## Data Availability

The datasets generated and/or analyzed during this study will be available upon reasonable request from the corresponding author.

## Abbreviations

Anti-Retroviral Therapy: ARV Adherence to Antiretroviral
CD4: Cluster of Differentiation 4
HIV: Human Immunodeficiency Virus
ICT: Information Communication Technology
LMIC: low middle-income country m-Health:Mobile Health
PLWHA: People Living With HIV/ADIS
SMS: Short Message Service
WHO: World Health Organization
